# Correlation between enlarged perivascular space and brain white matter hyperintensities in patients with recent small subcortical infarct

**DOI:** 10.1002/brb3.3168

**Published:** 2023-07-18

**Authors:** Xiuli Jian, Fubiao Xu, Mi Yang, Min Zhang, Wenwei Yun

**Affiliations:** ^1^ Department of Neurology Changzhou Second People's Hospital, Changzhou Medical Center, Nanjing Medical University Changzhou China; ^2^ Department of Cardiology Heze Municipal Hospital Heze China

**Keywords:** enlarged perivascular spaces, recent small subcortical infarct, semiautomatic quantitative evaluation, white matter hyperintensities

## Abstract

**Background:**

This study aimed to investigate the correlation between enlarged perivascular space (EPVS) and white matter hyperintensities (WMH) at different locations in patients with recent small subcortical infarct (RSSI).

**Methods:**

Data were collected from patients with RSSI who were hospitalized at Changzhou Second People's Hospital between October 2020 and December 2021. All patients underwent cranial magnetic resonance imaging, and the grades of EPVS and WMH were assessed, including basal ganglia EPVS (BG‐EPVS), centrum semiovale EPVS (CSO‐EPVS), deep WMH (DWMH), and periventricular WMH (PWMH). The volumes of EPVS and WMH at different locations were quantified using 3D Slicer software. Patients were grouped according to the severity of BG‐EPVS and CSO‐EPVS. Univariate and multivariate analyses were used to analyze the relationship between EPVS and WMH.

**Results:**

A total of 215 patients with RSSI were included in the analysis. Patients with moderate‐to‐severe BG‐EPVS had higher DWMH and PWMH severity than those with mild BG‐EPVS, both in terms of volume and grade. There was no significant difference in WMH severity between patients with mild CSO‐EPVS and those with moderate‐to‐severe CSO‐EPVS. Multivariate analysis indicated that after adjustments were made for confounding factors, DWMH volume (*β* = 0.311; 95% CI, 0.089–0.400; *p* = .002) and PWMH volume (*β* = 0.296; 95% CI, 0.083–0.424; *p* = .004) were independently associated with BG‐EPVS. Pearson correlation showed that PWMH volume (*r* = .589; *p* < .001) and DWMH volume (*r* = .596; *p* < .001) were positively related to BG‐EPVS volume.

**Conclusion:**

DWMH and PWMH are closely related to BG‐EPVS in patients with RSSI.

## Graphical abstract

Enlarged perivascular spaces and brain white matter hyperintensities are both imaging markers of cerebral small vascular disease. The relationship between enlarged perivascular spaces and brain white matter hyperintensities is unclear. This study aimed to investigate the correlation between EPVS and WMH at different locations in patients with recent small subcortical infarct.

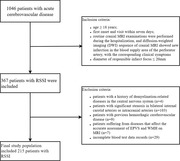

## 1 INTRODUCTION

The perivascular space (PVS) is a structure that surrounds the cerebral perforator arteries and small arteries, which play an essential role in the clearance of metabolites from the brain (Wardlaw et al., 2020). When the clearance of brain metabolic waste and fluid drainage is blocked, the PVS is pathologically expanded; this is known as enlarged PVS (EPVS) (Mestre et al., 2017). EPVS can be classified as basal ganglia EPVS (BG‐EPVS) or centrum semiovale EPVS (CSO‐EPVS) depending on location (Rudie et al., 2018). Previously, EPVS was considered to be an age‐related neurodegenerative lesion. However, recent studies have found that EPVS is a pathological dilation and is closely related to stroke prognosis, vascular dementia, cognitive dysfunction, gait abnormalities, and mood disorders (Arba et al., 2018; Yamaguchi et al., 2021). Therefore, it is necessary to understand the pathophysiology of EPVS and thus control its progression.

A white matter hyperintensity (WMH) is a small vessel lesion that is commonly seen in the elderly. Recently, the presence of WMH has attracted attention as a precursor to diseases such as stroke and dementia (Guo and Shi, 2022). WMH commonly occurs as a periventricular WMH (PWMH) or as a deep WMH (DWMH) (Wardlaw et al., 2013).

Several studies have demonstrated a positive correlation between EPVS and WMH severity (Debette et al., 2019; Liu et al., 2019; Potter et al., 2015). However, uncertainties regarding this association remain, such as the relationship between EPVS and WMH in various locations. Moreover, most of the studies that have evaluated EPVS and WMH have used visual assessment, which has poor accuracy and high subjectivity (Francis et al., 2019).

Recent small subcortical infarct (RSSI) is a new infarct that occurs within weeks in a single deep penetrating arterial supply area. The lesion typically has a diameter of **≤**20 mm on the diffusion sequence of magnetic resonance imaging (MRI) (Eppinger et al., 2019). The occurrence of RSSI accounts for approximately 30% to 35% of stroke cases; however, patients with RSSI usually have only mild clinical symptoms and small lesions that do not affect the imaging evaluation of EPVS and WMH (Cannistraro et al., 2019; Regenhardt et al., 2018). For this study, we therefore chose to enroll patients with RSSI so that we could visually assess the severity and quantitatively assess the volume of EPVS and WMH in a subset of patients with acute cerebrovascular disease. We analyzed the correlation between EPVS and WMH at various locations, aiming to provide some theoretical basis for a further understanding of the pathogenesis of EPVS.

## 2 METHODS

### 2.1 Study population

**FIGURE 1:**
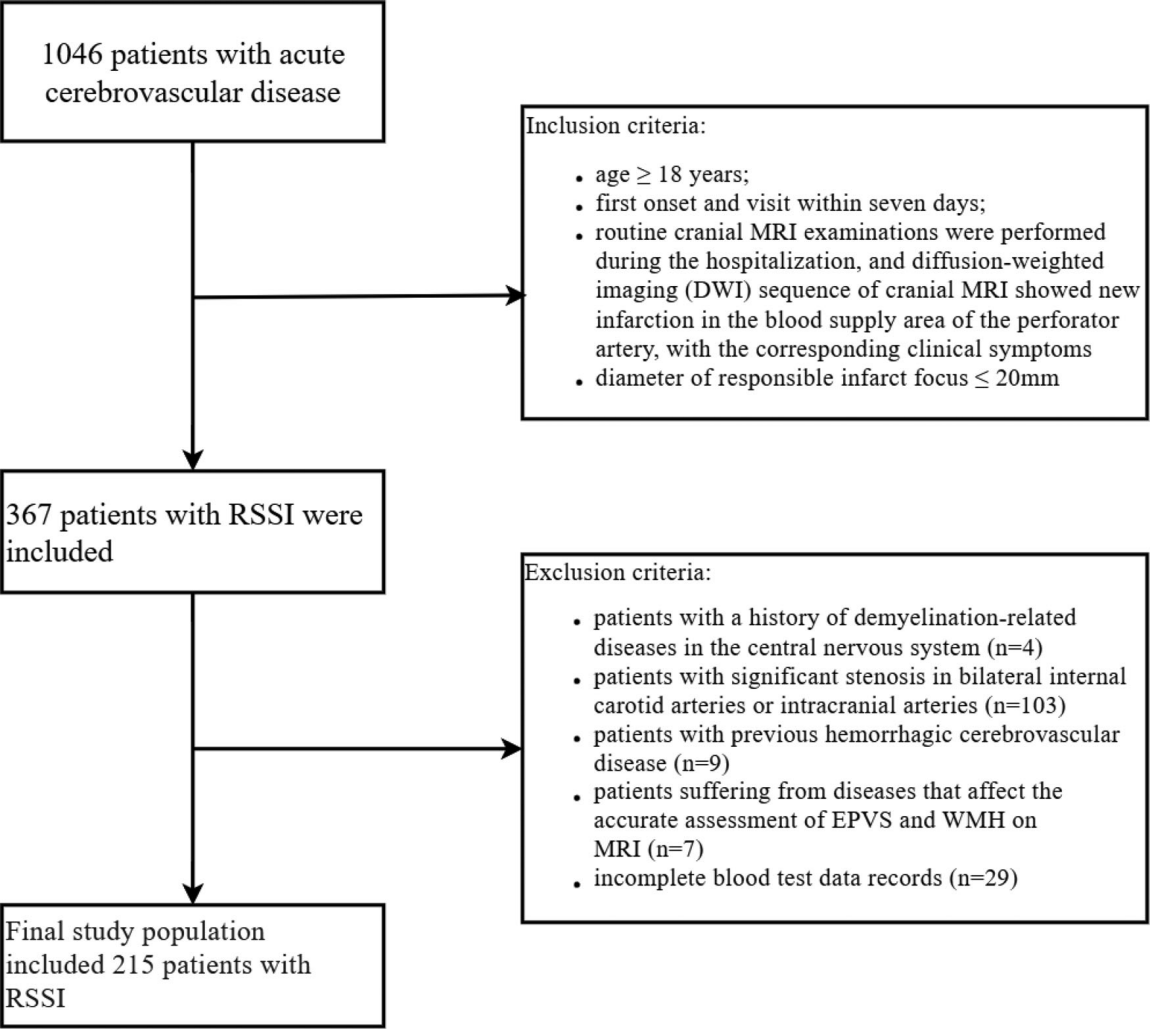
Flowchart demonstrating patient selection. EPVS, enlarged perivascular space; RSSI, recent small subcortical infarct; WMH, white matter hyperintensity.

Data from consecutive patients with acute cerebrovascular disease who were treated at Changzhou Second People's Hospital affiliated to Nanjing Medical University between October 2020 and December 2021 were collected retrospectively. Patients were eligible for study inclusion if they met the following criteria: (1) age ≥ 18 years; (2) hospital visit occurred within 7 days of first onset; (3) routine cranial MRI examinations were performed during the hospitalization, and diffusion‐weighted imaging (DWI) showed a new infarction in the blood supply area of the perforator artery, with corresponding clinical symptoms (Del Bene et al., 2013); and (4) diameter of responsible infarct focus was ≤20 mm. Patients were excluded from the analysis if they had any of the following: (1) history of demyelination‐related diseases in the central nervous system; (2) significant stenosis of the bilateral internal carotid arteries or intracranial arteries; (3) previous hemorrhagic cerebrovascular disease; (4) diseases that affected the accurate assessment of EPVS and WMH on MRI, such as severe hydrocephalus, intracranial infection, or history of brain trauma; and (5) incomplete blood test data (Figure 1). Finally, 215 RSSI patients were included.

This cross‐sectional study was approved by the Ethics Committee of Changzhou Second People's Hospital (batch No.: 2021KY312‐01). All enrolled patients or their family members provided written informed consent.

### 2.2 Data collection

General clinical data were collected from the medical records, including patient age; sex; body mass index; smoking status; alcohol use status; and history of diabetes, hypertension, and coronary heart disease. Relevant laboratory data were also collected, including levels of total cholesterol, triglycerides, low‐density lipoprotein cholesterol, high‐density lipoprotein cholesterol, fasting glucose, glycosylated hemoglobin, urea nitrogen, serum creatinine, uric acid, and homocysteine.

### 2.3 MRI acquisition and assessment

All MRI scans were performed on a 3.0 T system (Achieva 3.0; Philips, Royal Philips). All patients underwent cranial MRI examinations within 3 days of admission, including T1‐weighted imaging (T1WI), T2‐weighted imaging (T2WI), DWI, and fluid‐attenuated inversion recovery (FLAIR) imaging. The parameters were as follows: T1WI: repetition time (TR) = 1900 ms, echo time (TE) = 2.48 ms, flip angle = 9, field of view = 250 × 250 mm^2^, voxel size = 1 × 1 × 1 mm^3^, and 176 sagittal slices; T2WI: TR = 9000 ms, TE = 78 ms, echo train length = 180, field of view = 230 × 230 mm^2^, voxel size = 0.9 × 0.9 × 0.9 mm^2^, and 176 sagittal slices; DWI: TR = 8000 ms, TE = 97 ms, field of view = 220 × 220 mm^2^, voxel size = 2 × 2 × 2 mm^3^, and 76 sagittal slices; and FLAIR: TR = 8400 ms, TE = 94 ms, echo spacing = 4.24 ms, field of view = 230 × 230 mm^2^, echo train length = 220, voxel size = 1 × 1 × 1 mm^3^, and 176 sagittal slices. The total scan time was approximately 1 h. For each MRI examination, the brain's gray matter and white matter had to be distinguishable, the lesions had to be well‐defined. Two experienced neurologists blinded to the clinical information evaluated all scans to ensure the accuracy of results. When a disagreement occurred, another senior neurologist assessed the findings.

**FIGURE 2:**
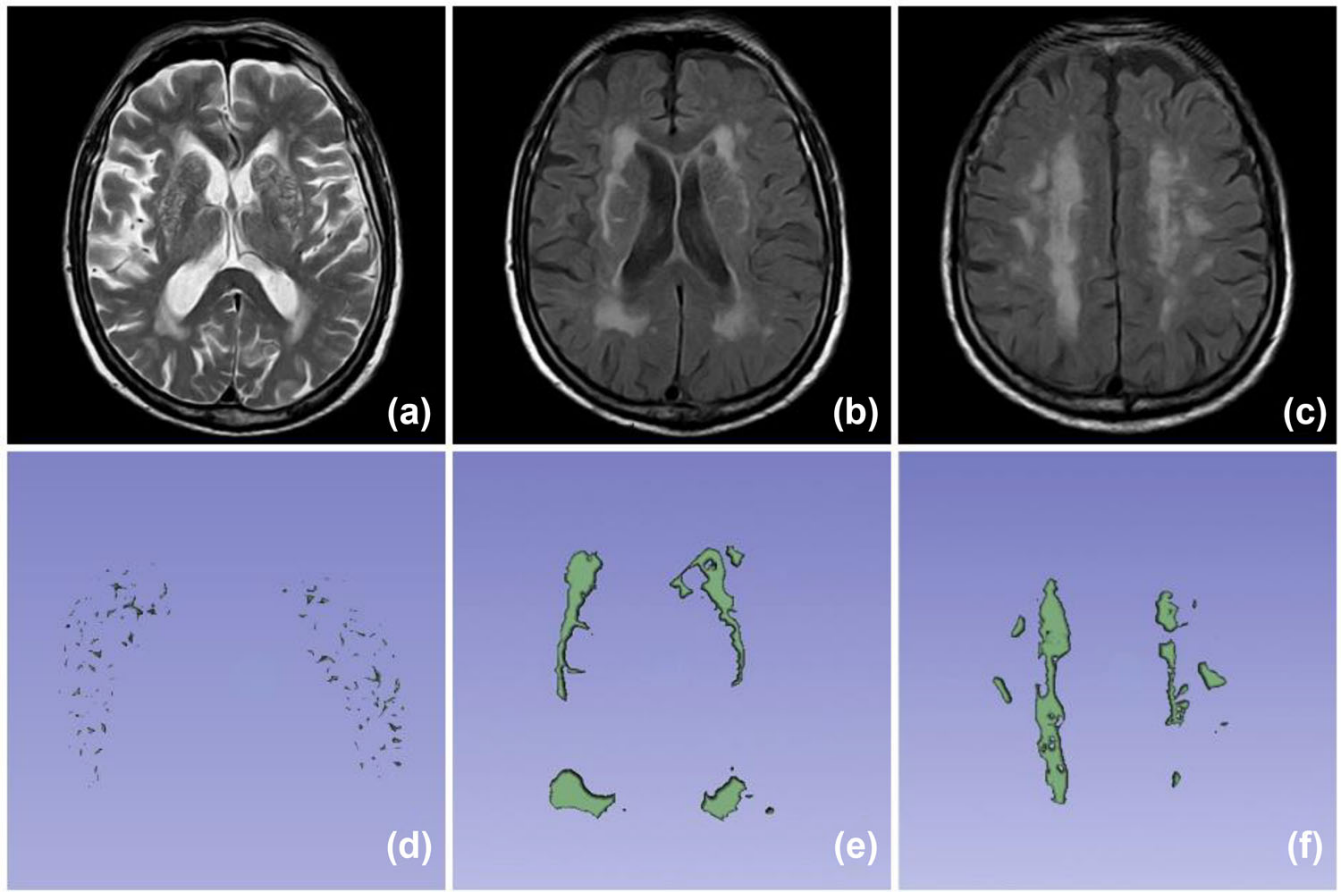
Cranial magnetic resonance (MR) images from a 73‐year‐old woman: (a) basal ganglia enlarged perivascular space (BG‐EPVS) grade was 4; (b) Fazekas score for periventricular white matter hyperintensity (PWMH) was 3; (c) Fazekas score for deep white matter hyperintensity (DWMH) was 3. (d)–(f) 3D reconstructed images of the scans shown in (a)–(c) (3D Slicer software).

For this study, EPVS was defined as an oval, linear, or tubular lesion that was consistent with the course of the perforator vessels and had a well‐defined border on MRI. The lesion had to be hypointense on T1WI and FLAIR imaging and hyperintense on T2WI, similar to the cerebrospinal fluid signal (Wardlaw et al., 2020). EPVS was further assessed with a combination of visual grading and semiautomatic quantitative evaluation on T2WI. The Potter score (Potter et al., 2015) was used to visually score the EPVS of both the BG and CSO sites separately. The level with the largest number of EPVS was selected, and if the left and right sides were asymmetrical, the side with more EPVS was selected. The grade was based on the number of EPVS as follows: grade 0, 0 EPVS; grade 1, 1–10 EPVS; grade 2, 11–20 EPVS; grade 3, 21–40 EPVS; and grade 4, >40 EPVS. Grades 0 and 1 were classified as mild EPVS, and grades 2–4 were classified as moderate‐to‐severe EPVS. EPVS volume was calculated semiautomatically on T2WI using 3D Slicer software. The process was as follows: First, the original MRI data were collected (DICOM format) and imported into the software program. Second, the editing mode was manually selected and the approximate threshold range of EPVS was set according to the difference in pixel signal intensity between the EPVS region and the normal region (Wang et al., 2016). Third, all EPVS at the level of the BG and CSO were marked layer by layer, and then the marked EPVSs were segmented. Finally, by running the model reconstruction mode, the segmented EPVSs were reconstructed in 3D, and the volumes of BG‐EPVS and CSO‐EPVS were calculated (Figure 2). In addition, ln‐transformation was performed on the raw volume data to reduce the effect of extreme data values and to improve data comparability.

For this study, WMH was defined as a high signal change with poorly defined margins in the periventricular or deep white matter on T2WI and FLAIR sequences. Visual grading was performed using the Fazekas scale to score the extent of PWMH and DWMH on the FLAIR sequence as follows: PWMH grade 0, no lesion; grade 1, hat‐like or pencillike thin‐layer lesions; grade 2, lesion presented as smooth halo; and grade 3, irregular paraventricular high signal extended to deep white matter; DWMH grade 0, no lesion; grade 1, punctate lesions; grade 2, lesions began to fuse; and grade 3, complete fusion of lesions area (Wardlaw et al., 2013). Evaluation of WMH volume at different locations was determined using 3D Slicer software (Figure 2). The raw volumetric data were ln‐transformed.

Because lacunes are closely related to EPVS and WMH, their presence or absence was also assessed. Lacunes were defined as round or oval lesions ranging from 3 to 15 mm in diameter, showing a low signal on T1WI, a high signal on T2WI, a high‐intensity edge, and a low signal in the center on FLAIR (Litak et al., 2020).

### 2.4 Statistical analysis

All statistical analyses were performed using the Windows SPSS software package (Version 26.0, IBM Corporation). Continuous variables with normal distribution were presented as mean ± SD, whereas continuous variables with skewed distribution were summarized as median and interquartile range. Comparisons between groups were performed using an independent sample *t*‐test or Mann–Whitney *U*‐test. Categorical variables were expressed as numbers and percentages. Comparisons between groups were performed using the chi‐square test. In the multivariate and correlation analyses, values with 0 BG‐EPVS and WMH volume were discarded to facilitate statistical analysis. Variables with *p* < .1 in the univariate analysis were included in the multiple linear regression analysis to explore the relationship between WMH volume and BG‐EPVS volume at different locations. The correlation between WMH volume and EPVS volume was tested using the Pearson rank method. *p* values < .05 were considered statistically significant.

## 3 RESULTS

### 3.1 Baseline characteristics of study patients

A total of 215 patients with RSSI were included in this study (mean age, 67 ± 12 years; 155 men, 60 women). A total of 164 study patients (76.3%) had hypertension. In addition, 90 patients (41.9%) had a history of diabetes, and 75 (34.9%) had a history of smoking. Lacunes were identified in 63 patients (29.3%).

### 3.2 Comparison of WMH severity in patients with mild EPVS and in those with moderate‐to‐severe EPVS

Of the 215 study patients, 122 patients (56.7%) had mild BG‐EPVS, and 93 patients (43.3%) had moderate‐to‐severe BG‐EPVS. There were significant differences in WMH grade and volume between the two groups for both DWMH and PWMH (*p* < .001 for all). Patients with moderate‐to‐severe BG‐EPVS had larger WMH volume and higher WMH grade than those with mild BG‐EPVS. Of note, patients with moderate‐to‐severe BG‐EPVS were also significantly older and more likely to have hypertension and lacunes than those with mild BG‐EPVS. Patients with moderate‐to‐severe BG‐EPVS also had higher urea nitrogen and homocysteine levels than those with mild BG‐EPVS (*p* < .05 for all). No significant differences were seen between the two groups in other clinical characteristics (Table 1).

**TABLE 1:** Comparison of patients with EPVS of different severities and locations.

Variable	Mild BG‐EPVS (*N* = 122)	Moderate‐to‐severe BG‐EPVS (*N* = 93)	*p* value	Mild CSO‐EPVS (*N* = 120)	Moderate‐to‐severe CSO‐EPVS (*N* = 95)	*p* value
Mean age ± SD, years	65 ± 12	70 ± 11	.001	68 ± 12	66 ± 12	.158
Male sex, *n* (%)	87 (71.3)	67 (72.0)	.906	91 (75.8)	63 (66.3)	.124
Mean body mass index ± SD	24.50 ± 3.59	23.93 ± 3.02	.223	24.13 ± 3.03	24.40 ± 3.74	.556
History of smoking, *n* (%)	45 (36.9)	30 (32.3)	.481	47 (39.2)	28 (29.5)	.139
History of alcohol use, *n* (%)	25 (20.5)	19 (20.4)	.991	30 (25.0)	14 (14.7)	.064
History of hypertension, *n* (%)	86 (70.5)	78 (83.9)	.022	83 (69.2)	81 (85.3)	.006
History of diabetes, *n* (%)	53 (43.4)	37 (39.8)	.590	45 (37.5)	45 (47.4)	.145
History of coronary heart disease, *n* (%)	6 (4.9)	8 (8.6)	.278	7 (5.8)	7 (7.4)	.651
Mean total cholesterol level ± SD, mmol/L	4.13 ± 1.04	4.25 ± 1.00	.375	4.15 ± 0.98	4.23 ± 1.06	.553
Median triglyceride level (IQR), mmol/L	1.49 (1.07, 2.02)	1.42 (1.06, 1.99)	.828	1.45 (1.06, 1.82)	1.50 (1.07, 2.13)	.245
Median LDL‐C level (IQR), mmol/L	2.06 (1.42, 2.74)	1.94 (1.16, 2.60)	.270	2.00 (1.24, 2.59)	2.02 (1.26, 2.79)	.767
Median HDL‐C level (IQR), mmol/L	1.16 (0.95, 1.60)	1.20 (0.98, 2.08)	.193	1.21 (0.94, 1.69)	1.16 (0.97, 1.61)	.537
Median HbA1c (IQR), %	6.20 (5.60, 8.05)	6.10 (5.60, 7.30)	.297	5.90 (5.60, 7.40)	6.30 (5.70, 8.03)	.106
Median fasting glucose level (IQR), mmol	5.62 (4.96, 7.64)	5.89 (5.04, 7.54)	.450	5.65 (4.83, 6.98)	6.00 (5.15, 8.34)	.063
Median urea nitrogen level (IQR), mmol/L	5.40 (4.40, 6.60)	6.00 (4.83, 7.15)	.016	5.50 (4.50, 6.90)	5.85 (4.75, 6.80)	.299
Median serum creatinine level (IQR), μmol/L	70.40 (62.00, 82.00)	73.00 (60.33, 91.00)	.433	72.00 (62.00, 88.50)	71.00 (60.15, 80.30)	.384
Mean uric acid level ± SD, μmol/L	318.87 ± 94.50	339.00 ± 90.48	.116	323.05 ± 92.58	323.28 ± 93.95	.425
Median homocysteine level (IQR), μmol/L	12.50 (9.68, 15.53)	13.20 (11.00, 18.15)	.010	13.35 (10.55, 15.95)	12.80 (9.80, 16.10)	.199
Presence of lacunes, *n* (%)	26 (21.3)	37 (39.8)	.003	24 (20.8)	39 (38.9)	<.001
PWMH grade, *n* (%)			<.001			.275
0	16 (13.1)	0 (0)		11 (9.2)	5 (5.3)	
1	89 (73.0)	30 (32.3)		60 (50.0)	59 (62.1)	
2	14 (11.5)	31 (33.3)		29 (24.2)	16 (16.8)	
3	3 (2.5)	32 (34.4)		20 (16.7)	15 (15.8)	
DWMH grade, *n* (%)			<.001			.325
0	20 (16.4)	0 (0)		14 (11.7)	6 (6.3)	
1	84 (68.9)	30 (32.3)		58 (48.3)	56 (58.9)	
2	9 (7.4)	21 (22.6)		19 (15.8)	11 (11.6)	
3	9 (7.4)	42 (45.2)		29 (24.2)	22 (23.2)	
Median PWMH volume (IQR), mm^3^	6.16 (5.57, 6.76)	8.01 (6.95, 8.50)	<.001	6.89 (6.04, 8.00)	6.57 (6.11, 8.11)	.805
Median DWMH volume (IQR), mm^3^	6.21 (5.69, 6.89)	7.99 (7.27, 8.34)	<.001	6.77 (5.79, 8.01)	6.72 (5.80, 8.01)	.694

Abbreviations: BG‐EPVS, basal ganglia enlarged perivascular space; CSO‐EPVS, centrum semiovale enlarged perivascular spaces; DWMH, deep white matter hyperintensity; HDL‐C, high‐density lipoprotein cholesterol; IQR, interquartile range; LDL‐C, low‐density lipoprotein cholesterol; PWMH, periventricular white matter hyperintensity; SD, standard deviation.

A total of 120 study patients (55.8%) had mild CSO‐EPVS, and 95 (44.2%) had moderate‐to‐severe CSO‐EPVS. Patients with moderate‐to‐severe CSO‐EPVS were more likely to have hypertension and lacunes than those with mild CSO‐EPVS (*p* < .05 for all). However, there were no significant differences in WMH grades or WMH volumes between the two groups (*p >* .05 for all) (Table 1).

### 3.3 Multiple linear regression correction analysis of WMH volume and BG‐EPVS volume

To facilitate multiple linear regression analysis, we discarded cases in which BG‐EPVS and WMH volumes were 0. The remaining BG‐EPVS volume was highly correlated with the BG‐EPVS grade (*r* = .922; *p* < .001). After multiple linear regression corrected for age, history of hypertension, urea nitrogen level, homocysteine level, and presence of lacunes, DWMH volume (*β* = 0.311; 95% CI, 0.089−0.400; *p* = .002) and PWMH volume (*β* = 0.296; 95% CI, 0.083−0.424; *p* = .004) were found to be independently associated with BG‐EPVS volume (Table 2).

**TABLE 2:** Multiple linear regression analysis of WMH volume and BG‐EPVS volume.

Variable	*β*	95% CI	*p* value
Age	0.022	−0.007 to 0.010	.730
History of hypertension	0.063	−0.112 to 0.371	.290
Urea nitrogen level	0.049	−0.035 to 0.080	.441
Homocysteine level	0.056	−0.005 to 0.015	.341
Presence of lacunes	0.036	−0.161 to 0.292	.567
PWMH volume	0.296	0.083 to 0.424	.004
DWMH volume	0.311	0.089 to 0.400	.002

Abbreviations: BG‐EPVS, basal ganglia enlarged perivascular space; CI, confidence interval; DWMH, deep white matter hyperintensity; PWMH, periventricular white matter hyperintensity.

### 3.4 Pearson correlation between WMH volume and BG‐EPVS volume

**FIGURE 3:**
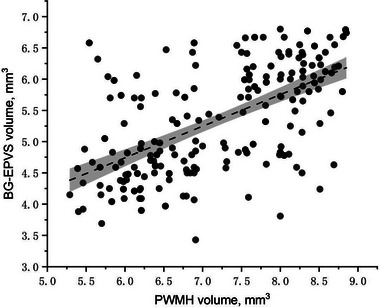
Pearson correlation analysis of basal ganglia enlarged perivascular space (BG‐EPVS) volume versus periventricular white matter hyperintensity (PWMH) volume in study patients (*r* = .589; *p* < .001).

**FIGURE 4:**
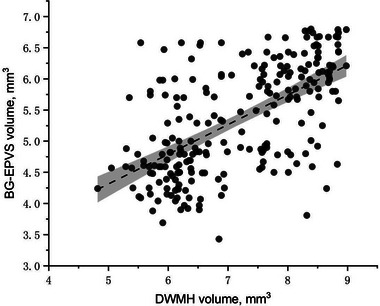
Pearson correlation analysis of basal ganglia enlarged perivascular space (BG‐EPVS volume) versus deep white matter hyperintensity (DWMH) volume in study patients (*r* = .596; *p* < .001).

Both PWMH volume (*r* = .589; *p* < .001) and DWMH volume (*r* = .596; *p* < .001) were positively correlated with BG‐EPVS volume (Figures 3 and 4).

## 4 DISCUSSION

In this study, patients with RSSI who had moderate‐to‐severe BG‐EPVS demonstrated more severe WMH (both in grade and volume) when compared with patients with mild BG‐EPVS. After corrections were made for confounding factors such as age, history of hypertension, and presence of lacunes, the volumes of PWMH and DWMH were found to be independently associated with BG‐EPVS volume. More specifically, the severity levels of PWMH and DWMH were found to be positively correlated with BG‐EPVS. However, PWMH and DWMH did not show a similar association with CSO‐EPVS.

Our findings are consistent with those of several previous studies that have shown a close association between WMH and EPVS, suggesting that WMH and EPVS may share the same pathophysiological mechanism (Doubal et al., 2010; Rouhl et al., 2008). However, these previous studies assessed only the grade of WMH and EPVS, not the volume. A more recent study used quantitative analysis software to calculate DWMH and EPVS volumes in 136 healthy participants and demonstrated a significant correlation between DWMH and EPVS volumes. In addition, this study also showed that free water might mediate this association, and the correlation between EPVS and WMH may be related to the damage of lymphatic drainage function and the accumulation of local interstitial fluid (Huang et al., 2021). Unfortunately, the study did not explore the correlation between PWMH and EPVS and did not classify EPVS based on location. We addressed these issues in the current study by subdividing WMH and EPVS according to location.

At present, the mechanism underlying the formation of cerebral small vascular disease is still unclear. Some scholars believe that WMH is caused by chronic hypoperfusion of the perforated arterial vessels. Inflammatory chain reaction, apoptosis, and blood–brain barrier disruption are involved in the formation of WMH (Bernbaum et al., 2015; Joutel and Chabriat, 2017; Swardfager et al., 2017). As for the pathogenesis of EPVS, several hypotheses have been proposed: (1) mechanical injury caused by cerebrospinal fluid pulsation or vasodilation; (2) ischemic injury secondary to perivascular tissue; and (3) segmental necrotizing vasculitis or other causes of increased permeability of the vessel wall, resulting in fluid accumulation in the tissue spaces (Del Brutto and Mera, 2018; Gouveia‐Freitas et al., 2021; Perosa et al., 2022). Among these hypotheses, increased permeability of the vessel wall is generally considered one of the most likely mechanisms. Research has also shown that inflammatory factors in the blood circulation are correlated with the formation of EPVS (Aribisala et al., 2014). Therefore, we hypothesize that EPVS and WMH might share a common pathological mechanism, both related to interstitial fluid leakage, functional disruption of the microvascular wall, and vascular inflammatory response.

A previous study in patients with acute ischemic stroke found that BG‐EPVS severity was associated with DWMH and PWMH, whereas CSO‐EPVS showed no significant association (Riba‐Llena et al., 2018). We reached similar conclusions in patients with RSSI, namely, that there was no significant difference in WMH volume or grade (DMWH or PWMH) between patients with mild CSO‐EPVS and those with moderate‐to‐severe CSO‐EPVS. This may be due to different PVS functions and mechanisms in different locations. Previous studies have shown that the anatomy differs between BG‐EPVS and CSO‐EPVS, with two layers of soft cerebral cells around the small blood vessels in the BG area and only one layer around the small blood vessels in the CSO area (van Leijsen et al., 2017; Wardlaw et al., 2020). According to the principle of arterial pulsation, the main driving force of cerebrospinal fluid flow in the PVS is in the BG area rather than in the center of the CSO area (Iliff et al., 2013). The pia mater is in the CSO area, and the superficial cortical vessels do not penetrate the subarachnoid space on the brain's surface.

BG‐EPVS is an imaging biomarker associated with hypertensive arterial disease and arteriosclerosis (Banerjee et al., 2017). WMH is also associated with hypertensive arteriopathy and arteriosclerosis. This may be one of the reasons why WMH is correlated with the severity of BG‐EPVS. In this study, patients with moderate‐to‐severe BG‐EPVS were more likely to have a history of hypertension than those with mild BG‐EPVS, which is consistent with previous studies. However, after multivariate linear regression analysis that adjusted for confounding factors, hypertension was not found to be independently associated with BG‐EPVS volume. This may be because most of these patients with RSSI were treated with blood pressure–lowering therapy. The severity and duration of the patient's hypertension may also affect the study results. Therefore, subsequent studies should record the severity of hypertension and the treatment of patients in detail.

Previous studies have used traditional visual grading to assess the severity of EPVS. This method is convenient and has few requirements in terms of technical conditions or hardware. However, visual grading is subjective, and differences are likely to occur among different assessors. Emerging quantitative evaluation methods based on digital image processing can greatly improve measurement accuracy and reduce the errors of visual analysis, leading to more objective and accurate imaging evaluation results. Currently, software analysis, convolutional neural network methods, deep machine learning, and other techniques can be used for the quantitative evaluation of EPVS (Dubost et al., 2019; Piantino et al., 2020; Williamson et al., 2022). In this study, we combined visual grading and quantitative assessment for a more precise assessment of EPVS and WMH, using the image analysis software 3D Slicer. Compared with the traditional visual grading method, this voxel‐based image extraction, segmentation, and calculation process can reduce the subjective influence of humans and achieve a more accurate evaluation of EPVS.

Our study has several potential limitations. This was a single‐center cross‐sectional study that could determine only correlation, not a causal relationship, between WMH and BG‐EPVS. Furthermore, the patients in this study had RSSI; the potential correlation between EPVS and WMH in a healthy population should be evaluated. Future studies will require increased sample sizes and cohort analysis to better assess these potential associations and to explore new ideas for delaying the development of cerebral small vessel disease and providing targeted treatment.

## 5 CONCLUSION

This study found that DWMH and PWMH were closely related to BG‐EPVS in patients with RSSI. The severity of WMH was directly proportional to the BG‐EPVS severity.

## CONFLICT OF INTERESTS STATEMENT

The authors have no financial interests related to this study.

### PEER REVIEW

The peer review history for this article is available at https://publons.com/publon/10.1002/brb3.3168

## Data Availability

The data that support the findings of this study are available from the corresponding author upon reasonable request.
